# Associations between the platelet/high-density lipoprotein cholesterol ratio and likelihood of nephrolithiasis: a cross-sectional analysis in United States adults

**DOI:** 10.3389/fendo.2024.1289553

**Published:** 2024-02-21

**Authors:** Junjie Ni, Lin Lv, Pu Wu, Chaoyang Xu

**Affiliations:** ^1^ Department of Breast and Thyroid Surgery, Affiliated Jinhua Hospital, Zhejiang University School of Medicine, Jinhua, Zhejiang, China; ^2^ Central Laboratory, Affiliated Jinhua Hospital, Zhejiang University School of Medicine, Jinhua, Zhejiang, China

**Keywords:** platelet/high-density lipoprotein cholesterol ratio, nephrolithiasis, NHANES, cross-sectional study, United States

## Abstract

**Aims:**

The primary objective of this study was to investigate the relationship between the platelet/high-density lipoprotein cholesterol ratio (PHR) and the prevalence of nephrolithiasis within the adult population of the United States.

**Methods:**

The data used in this study were obtained from the National Health and Nutrition Examination Survey (NHANES) conducted between 2007 and 2018. The analysis included a non-pregnant population aged 20 years or older, providing proper PHR index and nephrolithiasis data. The research utilized subgroup analyses and weighted univariate and multivariable logistic regression to evaluate the independent association between the PHR and the susceptibility to nephrolithiasis.

**Results:**

The study comprised 30,899 participants with an average PHR value of 19.30 ± 0.11. The overall prevalence rate of nephrolithiasis was estimated at 9.98% with an increase in the higher PHR tertiles (T1, 8.49%; T2, 10.11%; T3, 11.38%, *P* < 0.0001). An elevated PHR level was closely linked with a higher susceptibility to nephrolithiasis. Compared with patients in T1, and after adjusting for potential confounders in model 2, the corresponding odds ratio for nephrolithiasis in T3 was 1.48 (95% CI: 1.06 to 2.08), with a *P*-value = 0.02. The results of the interaction tests revealed a significant impact of chronic kidney disease on the relationship between PHR and nephrolithiasis. Furthermore, the restricted cubic spline analyses exhibited a positive, non-linear correlation between PHR and the risk of nephrolithiasis.

**Conclusion:**

A convenient biomarker, the PHR, was independently associated with nephrolithiasis and could be a novel biomarker in predicting occurrence in clinical decision.

## Introduction

Nephrolithiasis has become more common throughout the world in the last few years. The prevalence of this condition has notably risen within the United States (US). Specifically, the incidence rate has surged from 3.2% in the 1980s to 10.1% in 2016 (1, 2). The occurrence of symptomatic nephrolithiasis is particularly high among adult males (3). Kidney stone disease (KSD), a major health concern, is expected to affect more than 15% of males and over 5% of females by the age of 70 (4). Furthermore, recurrences are common in patients with symptomatic nephrolithiasis after the initial stone episode, with a recurrence rate of up to 50% over a lifetime in both genders (5, 6). Nephrolithiasis has garnered significant attention in healthcare due to its substantial medical costs and societal impact, with annual healthcare expenditures exceeding $2 billion in the US alone (7). Therefore, there is a pressing need to focus on the prevention of nephrolithiasis.

Despite the increasing prevalence of nephrolithiasis, various risk factors have been identified, including obesity, diabetes mellitus (DM), excessive consumption of salt, animal protein, and sugary foods (8–11). Recent research suggests that multiple inflammatory processes are involved in kidney stone formation. For example, the retention of crystals is mediated by the excessive production of reactive oxygen species, leading to subsequent oxidative stress and inflammation (12–14). During this inflammatory response, the levels of neutrophils, platelets (PLT), lymphocytes and acute phase proteins change. Among these, PLTs have been identified as crucial modulators of the inflammatory response and can also accelerate inflammatory state. Activated PLTs trigger the intrinsic coagulation cascade, contributing to multiple diseases (15). The platelet lymphocyte ratio (PLR) has been explored as an accurate marker for systematic inflammatory response syndrome (SIRS) in patients who underwent percutaneous nephrolithotomy (PCNL) (16). Moreover, high-density lipoprotein cholesterol (HDL-C) has demonstrated anti-platelet and anti-thrombotic properties (17, 18) and has also been identified as an anti-inflammatory group of proteins (19). Low levels of HDL-C have been associated with an increased risk of nephrolithiasis. Jialal et al. initially demonstrated a significant correlation between the platelet -to-HDL-C ratio (PHR) and the severity of Metabolic Syndrome (MetS) (20). These findings have led to speculation about using a combination of PLT and HDL-C levels to detect groups at high risk for nephrolithiasis earlier so that interventions can be implemented in advance to reduce the development of nephrolithiasis. However, there is limited knowledge regarding its predictive capacity for nephrolithiasis.

The current study utilized data on nephrolithiasis from the National Health and Nutrition Examination Survey (NHANES) to examine the association between PHR and the risk of kidney stone development in US adults.

## Materials and methods

### Study participants and data collection

**Figure 1:**
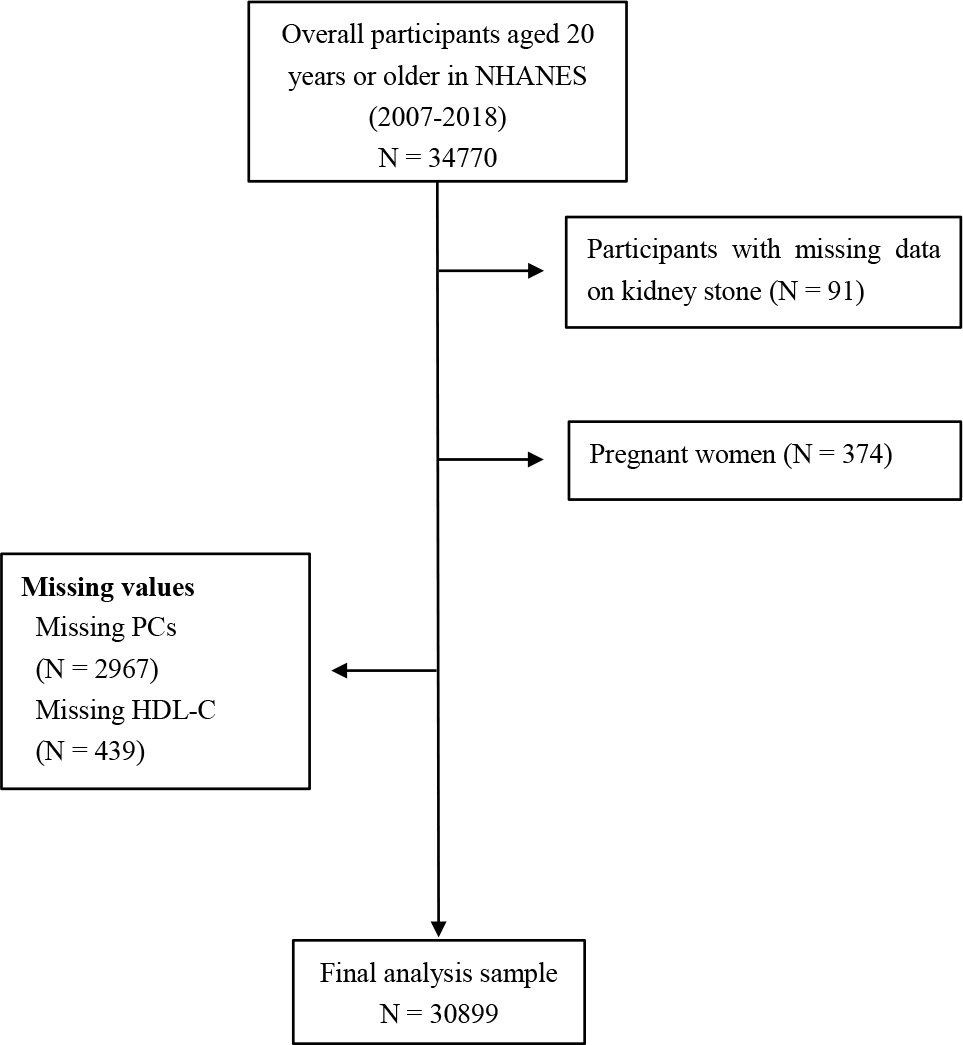
Representation of study design. NHANES, National Health and Nutrition Examination Survey; PCs, platelet counts; HDL-C, high-density lipoprotein cholesterol.

The data for this retrospective analysis were sourced from the NHANES, accessible via the official website of the Centers for Disease Control and Prevention (CDC, https://www.cdc.gov/nchs/nhanes/index.htm). The NHANES employs a systematic, multistage, probability-cluster method to collect data, which enables the assessment of health and nutritional trends in both adults and children in the US. The survey’s sampling strategy included an oversampling of participants from various racial and ethnic backgrounds such as non-Hispanic black, non-Hispanic white, Mexican American, etc. For the purposes of this study, NHANES data spanning from 2007 to 2018 were utilized. An initial review of the data for the specified study period provided records for 34,770 participants aged 20 years or older. However, the analysis excluded participants without nephrolithiasis data (n = 91), pregnant women (n = 374), those with missing platelet counts (PCs) (n = 2967), and those with missing HDL-C levels (n = 439). After applying the detailed inclusion and exclusion criteria, the study encompassed a total of approximately 30,899 participants (**Figure 1**). The NHANES protocol was approved by the National Center for Health Statistics Ethics Review Board, and the study was conducted in accordance with informed consent under ethical guidelines (21). The data utilized from the publicly available database was also openly accessible from other sources (22).

### Exposure variables and outcomes

In this study, the PHR was specifically constructed to function as the exposure variable. Due to the extensive variability in PHR values, a transformation of the PHR was applied. The PHR was calculated by dividing the PCs by the HDL-C levels and then dividing the quotient by 10 (20).

The primary outcome was determined based on the presence or absence of a self-reported history of kidney stones, covering both symptomatic and asymptomatic cases. This was ascertained by the participant’s response to the question, “Have you/Has sample person (SP) ever had a kidney stone?” Those who answered ‘yes’ were categorized as having nephrolithiasis. The validity of self-reported data on the occurrence of kidney stones has been confirmed by previous studies (4, 23, 24).

### Biochemical parameters

The study involved assessing various biochemical parameters in participants who voluntarily underwent blood sampling at a mobile examination center (MEC). The range of parameters measured included uric acid, fasting glucose, LDL-C, HDL-C, total cholesterol (TC), PCs, creatinine and blood urea nitrogen. The methodologies for these measurements were based on standardized procedures referenced from the current scientific literature (25, 26). For renal function assessment, the estimated glomerular filtration rate (eGFR) was calculated using the Chronic Kidney Disease Epidemiology Collaboration (CKD-EPI) equation based on creatinine levels, given by: eGFR = 141 × min(Scr/κ, 1)^α^ × max(Scr/κ, 1)^-1.209^ × 0.993^Age^ × 1.018 [if female] _ 1.159 [if black], where Scr is serum creatinine, κ is 0.7 for females and 0.9 for males, α is -0.329 for females and -0.411 for males, min indicates the minimum of Scr/κ or 1, and max indicates the maximum of Scr/κ or 1 (27).

### Other covariates of interest

In this study, an array of potential confounding variables identified from epidemiological research related to nephrolithiasis was examined. These included sociodemographic factors such as marital status, education level, race, age, and gender. Physical measures addressed were body mass index (BMI), while lifestyle factors taken into account encompassed alcohol consumption, smoking status and total plain water intake. Furthermore, the presence of comorbid conditions such as DM, hypertension, hyperlipidemia, CKD and coronary heart disease (CHD) was also investigated. In addition, indices such as the composite dietary antioxidant index (CDAI), dietary inflammation index (DII), and systemic immune-inflammatory index (SII) were calculated to assess their potential roles as moderating factors.

Sociodemographic data were gathered through self-reported questionnaires. Age was categorized as < 45, 45–64, or ≥ 65 years. Individuals’ sex was documented as either male or female. Race/ethnicity was classified as non-Hispanic black, non-Hispanic white, Mexican-American, or other, which included those with multiracial backgrounds. Marital status was recorded as never married, married, or living separately. Participants who were divorced, widowed, or living separately were classified as residing in separate households. Education level was classified into three categories: less than high school, high school graduate, or education beyond high school. According to the established criteria by the World Health Organization (WHO), individuals with a BMI equal to or exceeding 30 kg/m^2 are considered obese. The family poverty-to-income ratio (PIR), when less than 1.00, indicates that the household income is below the poverty threshold, while a PIR exceeding 3.00 signifies that the income is more than three times the poverty threshold.

The lifestyle factors were assessed using self-reported questionnaires. Alcohol consumption was quantified based on established criteria and participants were divided into four categories: lifetime abstainers, defined as those who have consumed fewer than 12 drinks in their lifetime; former drinkers, those who have had at least 12 drinks in their lifetime but have not consumed alcohol in the past year; current light drinkers, consuming three or fewer drinks per week; and current heavy drinkers, consuming more than three drinks per week. According to the National Center for Health Statistics and the CDC, participants’ smoking history was classified into three groups. The first group comprised individuals who had never smoked or had smoked fewer than 100 cigarettes in their lifetime. The second category included those who had smoked 100 or more cigarettes but had quit by the time of the interview. The third group included people who were presently smoking. Total plain water intake, measured in milliliters per day (ml/day), was defined as the total volume of water consumed from all sources—including plain tap water, water from drinking fountains or water coolers, bottled water, and spring water—over a 24 hours period.

This study also investigated the comorbidities, including hypertension, hyperlipidemia, DM, CKD and CHD. The inclusion criteria for hypertension included a medical diagnosis of hypertension, the use of anti-hypertensive medication, or a measured systolic blood pressure equal to or exceeding 140 mmHg and/or diastolic blood pressure equal to or exceeding 90 mmHg. Hyperlipidemia was defined by an HDL-C level less than 40 mg/dL, LDL-C level of 160 mg/dL or higher, triglycerides of 200 mg/dL or higher, and TC of 240 mg/dL or higher, or by a previous diagnosis obtained during the NHANES blood test. The participants were identified with type 2 DM based on specific criteria, including a medical diagnosis of diabetes, oral glucose tolerance test results of 11.1 mmol/L or higher, random glucose levels of 11.1 mmol/L or higher, fasting glucose levels of 7.0 mmol/L or higher, Hemoglobin A1c (HbA1c) of 6.5% or more, or the use of antidiabetic medication. The CKD-EPI equation was used to assess the eGFR for CKD diagnosis, with CKD defined as an eGFR of less than 60 ml/min/1.73 m^2, or a urine albumin-to-creatinine ratio (UACR) exceeding 30 mg/g (28). The presence of CHD was determined through self-reported data, with participants indicating ‘Yes’ or ‘No’.

Dietary data in NHANES were obtained through a 24-hour dietary recall interview and have been validated by the Nutrition Methodology Working Group (29). The CDAI measures an individual’s antioxidant profile by considering dietary antioxidants such as vitamins A, C, E, as well as selenium, zinc, and carotenoids; we calculated the CDAI as proposed by Wright et al. (30) The DII was designed to evaluate the inflammatory potential of diets based on the proinflammatory and anti-inflammatory properties of the dietary components (31). The detailed method for calculating the CDAI and DII have been described in previous studies (30, 31). The concept of the SII was first introduced by Hu et al. (32) to evaluate the prognostic value for multiple diseases. The SII is composed of counts of peripheral neutrophil (N), lymphocyte (L), and PLT, and is defined as PLT multiplied N/L (expressed in 10^9/L) (33).

### Statistical analysis

Data preparation and statistical analysis were carried out utilizing R software version 4.2 (http://www.R-project.org; The R Foundation). In accordance with NHANES recommendations, the study applied sampling weights to mitigate the purposeful oversampling of certain demographic categories. All tests were weighted for sample size and took into account the complex stratified, multistage, cluster sampling strategy used in NHANES (34). Continuous variables were represented as mean ± standard error (SE) with survey weights. Categorical variables were displayed as counts and percentages, adjusted for survey weights. The current study involved converting the variable corresponding to PHR from a continuous to a categorical scale. Subsequently, several models were developed to assess the individual impacts of PHR and nephrolithiasis on the outcome of interest. PHR was analyzed both as continuous and as categorical variables based on tertiles. Differences between groups, whether separated by PHR tertiles or by the presence versus absence of nephrolithiasis, were assessed using a weighted chi-square test for categorical variables and a weighted Student’s t-test for continuous variables.

The current study employed weighted univariate and multivariate logistic regression analyses to evaluate the relationship between PHR and nephrolithiasis across various models. Covariates with a *P* < 0.05 in the univariate analysis were included in the multivariate model as confounding factors. Crude Model did not incorporate flexible adjustments. Model 1 was adjusted for age, sex, and race. Model 2 included adjustments for age, sex, race, BMI, marital status, family PIR, smoking status, alcohol usage, hypertension, hyperlipidemia, CKD, CHD, DM, fasting glucose, uric acid, eGFR, creatinine, blood urea nitrogen, CDAI, DII and SII. The *P*-value for the trend was determined using a logistic regression model. Interaction analyses were also conducted to examine the heterogeneity of correlations among different subgroups. A restricted cubic spline (RCS) model with three knots was utilized to explore potential linear and non-linear relationships. Knot number 3 was selected by minimizing the Akaike information criterion (AIC) statistic. The log-likelihood ratio test was applied to assess the presence of linear or non-linear correlations. A *P*-value < 0.05 was considered statistically significant for all analyses.

## Results

### Personal characteristics of participants

The study included 30,899 participants, as indicated in **Table 1**. The mean age of the participants was 47.78 ± 0.23 years, and the population comprising 48.73% males and 51.27% females. The prevalence of nephrolithiasis accounted for 9.98% of the population. The average value of the PHR was 19.30 ± 0.11, and the observed PHR ranges for the three tertiles were as follows: tertile 1 ranged from 0.61 to 15.32, tertile 2 from 15.32 to 21.36, and tertile 3 from 21.36 to 183.52. The study observed that the individuals with higher PHR tertile tended to have a higher risk of nephrolithiasis (Tertile 1: 8.49%, Tertile 2: 10.11%, Tertile 3: 11.38%) (*P* < 0.0001). Significant variables across all tertiles of PHR included age, sex, ethnicity, marital status, educational level, household income, smoking status, alcohol use, BMI, PCs, fasting glucose, LDL-C, HDL-C, TC, uric acid, eGFR, blood urea nitrogen, CDAI, DII, SII, DM, hypertension, hyperlipidemia, CKD and CHD. Notably, participants in the higher PHR tertiles, compared to those in the lowest tertile, had increased levels of DII and SII and decreased levels of CDAI (*P* < 0.05). The clinical and biochemical characteristics of the participants with and without nephrolithiasis are shown in **Supplementary Table 1**. Cases of nephrolithiasis had higher levels of fasting glucose, uric acid, creatinine, blood urea nitrogen, DII, SII, and lower levels of eGFR, TC, and CDAI, and were more likely to be male, older, non-Hispanic White, married, more educated, of higher income, obese, non-drinkers, with DM, hypertension, hyperlipidemia, CKD and CHD (all *P* < 0.05).

**Table 1:** Study participants with clinical aspects.

Characteristics (weighted)		PHR categories			
	Total (N = 30,899)	T1 (< 15.32)	T2 (15.32–21.36)	T3 (21.36–183.52)	*P*-value
Platelet (1000cells/uL)	243.50 ± 0.82	203.11 ± 0.71	240.23 ± 0.63	288.60 ± 1.06	< 0.0001
TC (mmol/L)	4.99 ± 0.01	5.03 ± 0.02	4.96 ± 0.02	4.98 ± 0.02	0.002
HDL-C (mmol/L)	1.38 ± 0.01	1.74 ± 0.01	1.33 ± 0.00	1.06 ± 0.00	< 0.0001
LDL-C (mmol/L)	2.94 ± 0.01	2.83 ± 0.02	3.00 ± 0.02	3.01 ± 0.02	< 0.0001
Fasting glucose (mmol/L)	5.96 ± 0.02	5.72 ± 0.03	5.99 ± 0.03	6.23 ± 0.04	< 0.0001
Uric acid (umol/L)	323.20 ± 0.80	305.49 ± 1.12	324.22 ± 1.14	340.47 ± 1.40	< 0.0001
eGFR (ml/min/1.73m^2)	94.14 ± 0.31	91.26 ± 0.39	94.30 ± 0.36	96.95 ± 0.38	< 0.0001
Creatinine (umol/L)	78.37 ± 0.26	78.21 ± 0.39	78.60 ± 0.31	78.29 ± 0.44	0.67
Blood urea nitrogen (mmol/L)	4.93 ± 0.02	5.12 ± 0.03	4.90 ± 0.03	4.76 ± 0.03	< 0.0001
Total plain water (ml/day)	1155.25 ± 15.62	1170.73 ± 24.31	1130.81 ± 19.19	1164.65 ± 22.26	0.27
CDAI	0.81 ± 0.05	1.04 ± 0.07	0.84 ± 0.07	0.55 ± 0.07	< 0.0001
DII	1.40 ± 0.03	1.20 ± 0.04	1.38 ± 0.03	1.64 ± 0.04	< 0.0001
SII (10^9/L)	534.25 ± 3.49	444.79 ± 4.55	519.43 ± 4.39	641.98 ± 5.55	< 0.0001
Kidney stone					< 0.0001
No	27953(90.02)	9432(91.51)	9303(89.89)	9218(88.62)	
Yes	2946(9.98)	869(8.49)	990(10.11)	1087(11.38)	
Age (years, n (%))					< 0.0001
<45	12670(44.72)	3447(37.21)	4285(46.23)	4938(50.92)	
45-64	10755(36.48)	3540(37.27)	3575(35.66)	3640(36.52)	
>=65	7474(18.80)	3314(25.52)	2433(18.11)	1727(12.56)	
Sex, n (%)					< 0.0001
Male	15133(48.73)	4507(40.61)	5194(51.44)	5432(54.34)	
Female	15766(51.27)	5794(59.39)	5099(48.56)	4873(45.66)	
Race, n (%)					< 0.0001
Mexican American	4706(8.63)	1163(5.97)	1614(8.90)	1929(11.12)	
Non-Hispanic Black	6355(10.68)	2499(11.87)	2059(10.41)	1797(9.73)	
Non-Hispanic White	12785(66.72)	4375(69.67)	4274(66.94)	4136(63.44)	
Other race	7053(13.97)	2264(12.49)	2346(13.76)	2443(15.71)	
Marital status, n (%)					< 0.0001
Married	18336(63.26)	5876(63.07)	6196(62.95)	6264(63.79)	
Live separated	6947(18.57)	2622(20.25)	2200(17.94)	2125(17.48)	
Never married	5601(18.13)	1798(16.65)	1892(19.06)	1911(18.72)	
Missing	15(0.03)	5(0.03)	5(0.05)	5(0.02)	
Education level, n (%)					< 0.0001
Less than high school	3295(5.48)	991(4.75)	1097(5.41)	1207(6.30)	
High school	11336(33.36)	3394(28.69)	3811(33.09)	4131(38.45)	
More than high school	16233(61.10)	5903(66.47)	5376(61.45)	4954(55.18)	
Missing	35(0.07)	13(0.09)	9(0.05)	13(0.06)	
Family PIR, n (%)					< 0.0001
< 1	6038(13.42)	1722(11.78)	2012(14.56)	2304(17.28)	
1-3	11936(33.43)	3829(33.43)	3924(36.19)	4183(38.97)	
> 3	10023(45.57)	3751(54.78)	3401(49.25)	2871(43.75)	
BMI (kg/m^2, n (%))					< 0.0001
<30	8708(29.12)	4332(44.42)	2745(27.60)	1631(15.75)	
>=30	21800(69.89)	5842(55.58)	7435(72.40)	8523(84.25)	
Smoking status, n (%)					< 0.0001
Never	17190(55.62)	5988(58.20)	5786(56.47)	5416(52.09)	
Former	7418(24.66)	2597(26.35)	2520(24.81)	2301(22.76)	
Now	6271(19.68)	1707(15.40)	1984(18.71)	2580(25.09)	
Missing	20(0.04)	9(0.06)	3(0.01)	8(0.05)	
Alcohol usage, n (%)					< 0.0001
Never	3950(9.86)	1303(9.63)	1308(9.76)	1339(10.19)	
Former	4296(11.44)	1227(8.86)	1424(11.64)	1645(13.91)	
Moderate	6736(25.45)	2703(32.20)	2163(23.94)	1870(20.02)	
Heavy	5540(19.33)	1699(18.29)	1821(19.04)	2020(20.71)	
Missing	10377(33.92)	3369(31.03)	3577(35.61)	3431(35.17)	
DM, n (%)					< 0.0001
No	24817(85.27)	8625(88.83)	8318(85.96)	7874(80.86)	
Yes	6082(14.73)	1676(11.17)	1975(14.04)	2431(19.14)	
Hypertension, n (%)					0.01
No	18021(63.31)	5941(64.73)	6168(64.48)	5912(60.64)	
Yes	12877(36.69)	4360(35.27)	4124(35.52)	4393(39.36)	
Missing	1(0.00)	0(0.00)	1(0.00)	0(0.00)	
Hyperlipidemia, n (%)					< 0.0001
No	8698(29.29)	4104(40.50)	3246(33.64)	1348(13.22)	
Yes	22201(70.71)	6197(59.50)	7047(66.36)	8957(86.78)	
CKD, n (%)					< 0.0001
No	24839(84.33)	8140(83.03)	8469(86.07)	8230(83.88)	
Yes	5665(14.56)	2035(16.06)	1707(12.92)	1923(14.71)	
Missing	395(1.11)	126(0.92)	117(1.01)	152(1.41)	
CHD, n (%)					0.03
No	29512(96.25)	9789(95.89)	9816(96.22)	9907(96.66)	
Yes	1274(3.51)	478(3.92)	444(3.55)	352(3.03)	
Missing	113(0.24)	34(0.19)	33(0.23)	46(0.31)	

Continuous data are shown as means and standard error (SE), while categorical data are presented as percentages.

LDL-C, low-density lipoprotein cholesterol; HDL-C, high-density lipoprotein cholesterol; TC, total cholesterol; PIR, poverty income ratio; BMI, body mass index; PHR, platelet/high-density lipoprotein cholesterol ratio; CKD, chronic kidney disease; DM, diabetes mellitus; eGFR, estimated glomerular filtration rate; CHD, coronary heart disease; CDAI, composite dietary antioxidant index; DII, dietary inflammation index; SII, systemic immune-inflammatory index.

### Potential link between PHR and nephrolithiasis risk

As shown in **Table 2**, the results of the univariate analysis indicated that PHR, age, non-Hispanic White, having a BMI ≥ 30 kg/m^2, a family PIR of 1-3, history of smoking, former alcohol use, and a history of DM, hypertension, hyperlipidemia, CKD, CHD, high levels of fasting glucose, uric acid, creatinine, blood urea nitrogen, DII and SII were all positively associated with nephrolithiasis (*P* < 0.05). Conversely, being female, non-Hispanic Black, never married, heavy alcohol use, higher eGFR, and higher CDAI, were negatively associated with nephrolithiasis (*P* < 0.01).

**Table 2:** Univariate logistic regression analysis of various variables.

Variables	OR (95% CI)	*P*-value
Age (versus <45, years)		
45-64	1.89(1.66,2.16)	<0.0001
>=65	2.25(2.00,2.52)	<0.0001
Sex (versus Male)		
Female	0.76(0.68,0.85)	<0.0001
Race (versus Mexican American)		
Non-Hispanic Black	0.73(0.62,0.86)	<0.001
Non-Hispanic White	1.70(1.49,1.94)	<0.0001
Other race	1.15(0.95,1.38)	0.15
BMI (versus < 30, kg/m^2)		
>=30	1.80(1.59,2.04)	<0.0001
Marital status (versus Married)		
Live separated	1.06(0.94,1.21)	0.34
Never married	0.48(0.41,0.57)	<0.0001
Missing	0.55(0.11,2.72)	0.46
Education level (versus Less than high school)		
High school	1.08(0.89,1.31)	0.42
More than high school	1.05(0.88,1.25)	0.59
Missing	0.80(0.15,4.33)	0.79
Family PIR (versus < 1)		
1-3	1.16(1.03,1.31)	0.02
> 3	1.13(0.98,1.31)	0.10
Smoking status (versus Never)		
Former	1.41(1.23,1.62)	<0.0001
Now	1.12(0.98,1.27)	0.08
Missing	0.59(0.13,2.70)	0.49
Alcohol usage (versus Never)		
Former	1.43(1.16,1.75)	<0.001
Moderate	0.89(0.70,1.12)	0.31
Heavy	0.71(0.57,0.88)	0.003
Missing	1.09(0.89,1.35)	0.40
DM (versus No)		
Yes	2.13(1.89,2.40)	<0.0001
Hypertension (versus No)		
Yes	1.96(1.75,2.18)	<0.0001
Missing	0.00(0.00,0.01)	<0.0001
Hyperlipidemia (versus No)		
Yes	1.64(1.44,1.87)	<0.0001
CKD (versus No)		
Yes	1.70(1.50,1.93)	<0.0001
Missing	1.14(0.73,1.80)	0.56
CHD (versus No)		
Yes	2.10(1.67,2.64)	<0.0001
Missing	1.83(1.01,3.35)	0.05
Total plain water (ml/day)	1.00(1.00,1.00)	0.31
PHR continuous	1.01(1.01,1.02)	<0.0001
TC (mmol/L)	0.98(0.92,1.03)	0.40
LDL-C (mmol/L)	1.00(0.92,1.08)	0.92
Fasting glucose (mmol/L)	1.11(1.08,1.13)	<0.0001
Uric acid (umol/L)	1.00(1.00,1.00)	<0.0001
eGFR (ml/min/1.73m^2)	0.99(0.98,0.99)	<0.0001
Creatinine (umol/L)	1.00(1.00,1.00)	<0.0001
Blood urea nitrogen (mmol/L)	1.10(1.08,1.13)	<0.0001
CDAI	0.98(0.97,1.00)	0.01
DII	1.06(1.03,1.09)	<0.001
SII (10^9/L)	1.00(1.00,1.00)	<0.001

LDL-C, low-density lipoprotein cholesterol; HDL-C, high-density lipoprotein cholesterol; TC, total cholesterol; PIR, poverty income ratio; BMI, body mass index; PHR, platelet/high-density lipoprotein cholesterol ratio; CKD, chronic kidney disease; DM, diabetes mellitus; eGFR, estimated glomerular filtration rate; CHD, coronary heart disease; CDAI, composite dietary antioxidant index; DII, dietary inflammation index; SII, systemic immune-inflammatory index; OR, odds ratio; CI, confidence interval.

The results of the multivariate logistic regression analysis demonstrated a significant relationship between the exposure and outcome variables, which persisted after adjusting for confounding factors (*P-*value < 0.05). The findings from the multivariate regression analyses are presented in **Table 3**. When treated as a continuous variable, PHR levels were positively associated with nephrolithiasis in adjusted model 2 (odds ratio [OR] = 1.02, 95% confidence interval [CI]: 1.00 – 1.04, *P* = 0.01). As categorical variables (divided into tertiles), the highest tertile of PHR was also positively associated with nephrolithiasis compared to the lowest tertile in adjusted model 2 (OR = 1.48, 95% CI: 1.06 – 2.08, *P* = 0.02). The *P*-value for the trend across tertiles was 0.02 in adjusted model 2.

**Table 3:** Relationship between the risk of nephrolithiasis and PHR *via* weighted multivariable logistic regression.

	Crude model		Model 1		Model 2	
	OR (95%CI)	*P*-value	OR (95%CI)	*P*-value	OR (95%CI)	*P*-value
**PHR continuous**	1.01(1.01,1.02)	<0.0001	1.02(1.01,1.02)	<0.0001	1.02(1.00,1.04)	0.01
PHR categories						
T1	ref		ref		ref	
T2	1.21(1.05,1.40)	0.01	1.27(1.10,1.48)	0.002	1.14(0.86,1.52)	0.34
T3	1.38(1.21,1.59)	<0.0001	1.52(1.33,1.74)	<0.0001	1.48(1.06,2.08)	0.02
*P* for trend		<0.0001		<0.0001		0.02

Crude model: PHR (platelet/high-density lipoprotein cholesterol ratio). Model 1: PHR, age, sex, race. Model 2: PHR, age, sex, race, BMI (body mass index), marital status, education level, family PIR (poverty income ratio), smoking status, DM (diabetes mellitus), alcohol consumption, hypertension, hyperlipidemia, CKD (chronic kidney disease), TC (total cholesterol), LDL-C (low-density lipoprotein cholesterol), fasting glucose, uric acid, eGFR (estimated glomerular filtration rate), creatinine, blood urea nitrogen, CDAI (composite dietary antioxidant index), DII (dietary inflammation index), SII (systemic immune-inflammatory index). OR; odds ratio, CI; confidence interval, Ref; reference.

**Figure 2:**
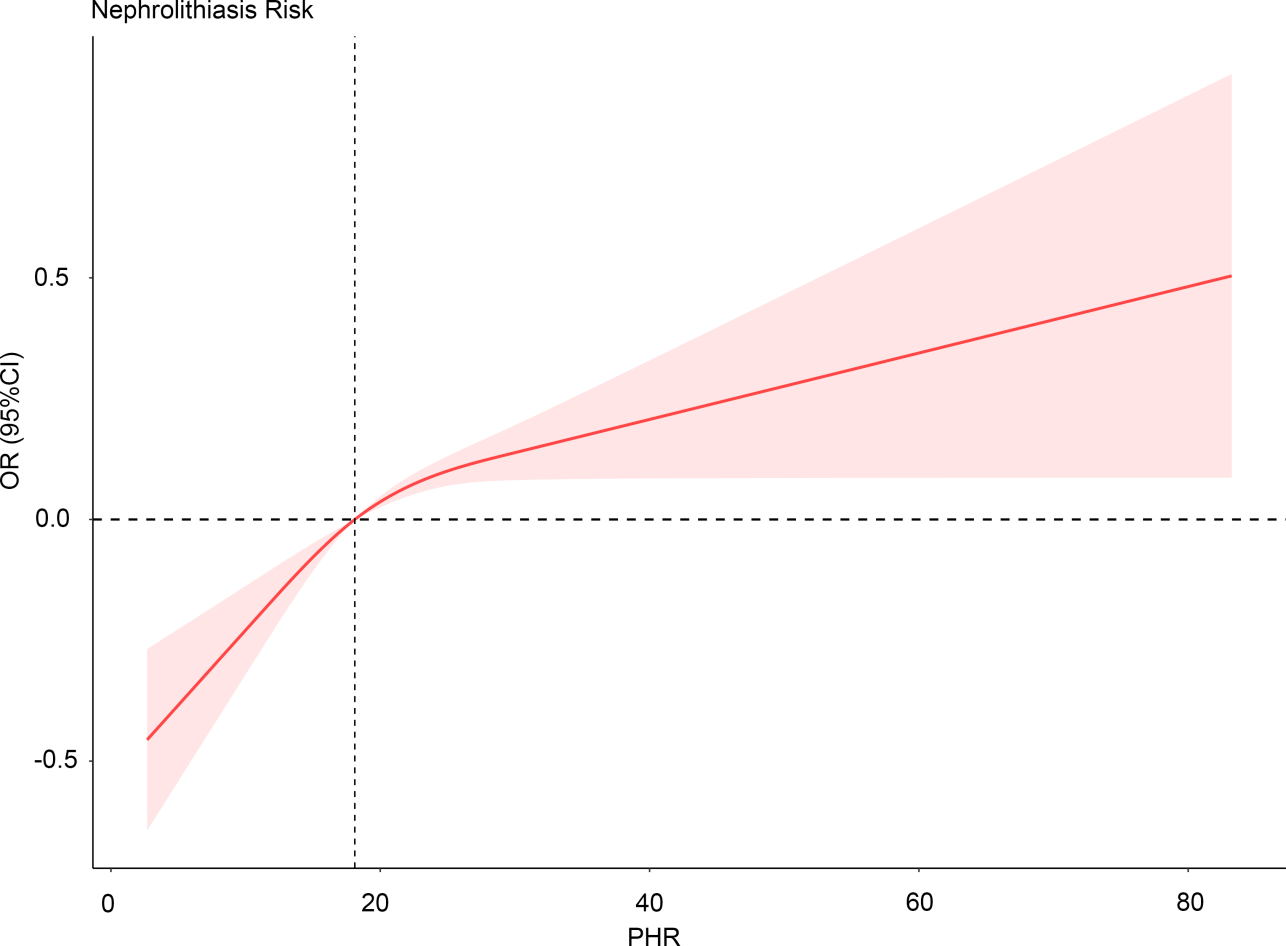
Restricted cubic spline analysis of the association between PHR values and the prevalence of nephrolithiasis. No variables have been adjusted. PHR, platelet/high-density lipoprotein cholesterol ratio; OR, odds ratio; CI, confidence interval; Ref, reference.

Additionally, the RCS analysis revealed a positive non-linear relationship between the risk of nephrolithiasis and PHR levels (*P* for nonlinearity = 0.0061), as illustrated in **Figure 2**.

### Stratified analyses

As shown in **Table 4**, a subgroup analysis stratified by various variables was conducted. Across all subgroups, participants in tertile 3 consistently had higher risks of nephrolithiasis compared to those in tertile 1 (all OR > 1). However, the association was not statistically significant in some subgroups. Notably, in the BMI-stratified analysis, the relationship between increased PHR levels and elevated risk of nephrolithiasis was significant for individuals with BMI ≥ 30 kg/m^2, with a 20.1% increase in risk for the T3 group compared to the T1 group, but this increase was not significant for individuals with BMI < 30 kg/m^2. In the analysis stratified by alcohol usage, there was a significant 55.5-89.7% increase in the risk of nephrolithiasis among moderate to heavy alcohol users; however, this increase was not significant in the never or former alcohol usage subgroup. In the CHD-stratified analysis, there was a significant 39.2% increase in the risk of nephrolithiasis in the group without CHD, but this increase was not significant in the CHD subgroup.

**Table 4:** Subgroup analyses of the relationship between PHR values and the prevalence of nephrolithiasis.

Characteristics	T1 (< 15.32)	T2 (15.32–21.36)	T3 (21.36–183.52)	*P* for trend	*P* for interaction
Age (years)					0.071
<45	ref	1.339(0.990,1.811)	1.884(1.456,2.439)	<0.0001	
45-64	ref	1.296(1.053,1.595)	1.535(1.244,1.893)	<0.0001	
>=65	ref	1.356(1.074,1.712)	1.242(1.004,1.536)	0.015	
Sex					0.672
Male	ref	1.205(0.996,1.458)	1.298(1.075,1.567)	0.008	
Female	ref	1.147(0.913,1.442)	1.394(1.144,1.699)	0.001	
Race					0.278
Mexican American	ref	0.933(0.658,1.322)	1.004(0.719,1.402)	0.91	
Non-Hispanic Black	ref	1.047(0.772,1.418)	1.008(0.726,1.401)	0.945	
Non-Hispanic White	ref	1.253(1.042,1.507)	1.476(1.248,1.746)	<0.0001	
Other race	ref	1.332(0.968,1.833)	1.592(1.201,2.110)	0.002	
Education level					0.4
Less than high school	ref	1.070(0.723,1.584)	1.511(1.012,2.257)	0.038	
High school	ref	1.098(0.881,1.369)	1.187(0.975,1.444)	0.085	
More than high school	ref	1.280(1.044,1.570)	1.491(1.217,1.827)	<0.001	
Marital status					0.96
Married	ref	1.240(1.031,1.491)	1.447(1.212,1.727)	<0.0001	
Live separated	ref	1.208(0.929,1.572)	1.318(1.073,1.619)	0.009	
Never married	ref	1.264(0.860,1.857)	1.347(0.920,1.971)	0.121	
Family PIR					0.068
< 1	ref	1.232(0.929,1.633)	1.766(1.382,2.257)	<0.0001	
1-3	ref	1.037(0.851,1.263)	1.171(0.971,1.414)	0.093	
> 3	ref	1.400(1.100,1.782)	1.567(1.240,1.980)	<0.001	
BMI (kg/m^2)					0.919
<30	ref	1.157(0.865,1.548)	1.180(0.841,1.655)	0.261	
>=30	ref	1.098(0.929,1.297)	1.201(1.032,1.397)	0.016	
Smoking status					0.484
Never	ref	1.221(0.993,1.501)	1.433(1.191,1.725)	<0.001	
Former	ref	1.362(1.080,1.717)	1.369(1.056,1.775)	0.015	
Now	ref	0.978(0.674,1.420)	1.285(0.980,1.686)	0.036	
Alcohol usage					0.566
Never	ref	0.993(0.645,1.529)	1.165(0.704,1.927)	0.546	
Former	ref	1.153(0.804,1.653)	1.245(0.890,1.740)	0.202	
Moderate	ref	1.134(0.835,1.539)	1.555(1.139,2.122)	0.008	
Heavy	ref	1.448(0.975,2.152)	1.897(1.295,2.781)	0.001	
DM					0.481
No	ref	1.142(0.961,1.357)	1.295(1.092,1.536)	0.003	
Yes	ref	1.357(1.058,1.741)	1.324(1.036,1.692)	0.054	
Hypertension					0.821
No	ref	1.167(0.946,1.441)	1.340(1.107,1.623)	0.003	
Yes	ref	1.265(1.048,1.525)	1.363(1.137,1.633)	0.001	
Hyperlipidemia					0.349
No	ref	1.237(0.976,1.568)	1.041(0.727,1.491)	0.415	
Yes	ref	1.159(0.979,1.373)	1.262(1.088,1.463)	0.002	
CKD					0.005
No	ref	1.117(0.946,1.318)	1.327(1.143,1.539)	<0.001	
Yes	ref	1.862(1.466,2.365)	1.757(1.304,2.368)	<0.001	
CHD					0.548
No	ref	1.198(1.031,1.391)	1.392(1.206,1.606)	<0.0001	
Yes	ref	1.523(0.997,2.328)	1.460(0.937,2.274)	0.073	

PHR, platelet/high-density lipoprotein cholesterol ratio; BMI, body mass index; PIR, poverty income ratio; DM, diabetes mellitus; CKD, chronic kidney disease; CHD, coronary heart disease; OR, odds ratio; CI confidence interval; Ref, reference.

The interaction test indicated that, with the exception of CKD (*P* interaction < 0.05), there were no significant differences among the other stratifications in the association between PHR and nephrolithiasis prevalence. These results suggest that the positive association between PHR and the risk of nephrolithiasis was consistent across populations differing in alcohol usage, Family PIR, marital status, age, gender, race, education level, BMI, smoking status, DM, hypertension, hyperlipidemia and CHD (all *P* for the interaction > 0.05), indicating that the findings could be applicable in various population settings.

## Discussion

In this observational study, we analyzed standardized data from a large cohort of participants in a US population sample. Our study found that the PHR was higher in patients with nephrolithiasis. Considering that an imbalance in the baseline characteristics of participants could modify the association between PHR and nephrolithiasis, adjustments were made for potential confounders in the regression analysis; nevertheless, we still observed a significant association of PHR with nephrolithiasis. This suggests that the association cannot be solely attributed to known risk factors and that PHR could independently predict the presence of nephrolithiasis. According to the subgroup analysis and interaction test, this connection was consistent across diverse demographic settings. In the RCS analysis, PHR demonstrated a pronounced non-linear association with the risk of nephrolithiasis, providing substantial evidence for further clinical and basic research.

This investigation reported the novel findings by representing the first examination of the correlation between PHR and nephrolithiasis. Researchers have recently suggested that kidney stone formation entails activating various inflammatory responses. We found that participants with kidney stones had lower levels of CDAI and higher levels of DII and SII, indicating that antioxidants, oxidants, and a pro-inflammatory diet may play a critical role in kidney stone formation. Dietary intake is an important source of antioxidants and oxidants. According to a study by Maddahi et al. (35), consuming a diet with an elevated DII is associated with a higher susceptibility to kidney stone formation in men. In another study, Zhang et al. (36) observed a positive association between an increased intake of a pro-inflammatory diet and a greater risk of kidney stone occurrence and recurrence. The DII provides a method for assessing the impact of diet on inflammation. However, anti-inflammatory diets, including the Dietary Approaches to Stop Hypertension (DASH) and the Mediterranean diet, may decrease systemic inflammation (37, 38). The regulation of inflammation requires various anti-inflammatory metabolites derived from dietary components, particularly short-chain fatty acids, tryptophan metabolites, and tyrosine metabolites, which have been identified as critical in the regulation of inflammation. Conversely, diets with a higher DII may potentially increase the overall level of systemic inflammation, which is involved in the development of nephrolithiasis. Cohen et al. (39) observed a potential beneficial effect of statin consumption on the development of urolithiasis. However, the precise mechanism remains elusive, and preliminary evidence suggests that statins possess anti-inflammatory and antioxidant properties that could potentially inhibit the incidence of nephrolithiasis. Elevated inflammation creates a conducive environment for the development and accumulation of renal tubular crystals, as verified in a murine model (40). Using statin can effectively reduce inflammatory mediators, thus inhibiting the retention of renal crystals (41). However, there are few studies on the role of immune responses and inflammatory cells in the formation of kidney stones. Idiopathic calcium oxalate stones often attach to Randall’s plaque, which are associated with the activation of M1 macrophages (42). While M2 macrophage-related genes are associated with the inhibition of stone formation (43). The renal crystal deposition is also related to the production of reactive oxygen species (ROS) production and inflammasome activation (44). Exosomes released by macrophage promote IL-8 production, facilitate neutrophil migration, and enhance the crystal inflammatory response (45). PLT interact with monocytes, neutrophils, and lymphocytes and regulate innate and adaptive responses. Thus, the imbalance of PHR may originate from the body’s immune and inflammatory response and may indirectly reflect the potential for kidney stone formation. However, the specific mechanistic relationship between PHR imbalance and nephrolithiasis formation needs further studied.

Furthermore, the risk of nephrolithiasis may be heightened by low levels of HDL-C. Torricelli et al. (46) reported that a significant correlation between reduced HDL-C levels and lower urinary pH. Moreover, a decreased urinary pH is implicated in the development of nephrolithiasis, particularly those characterized by uric acid stones (47–49). A urinary pH below 5.5 significantly increases the concentration of soluble non-dissociated uric acid, leading to the precipitation and formation of uric acid stones (47, 50). The primary concern stems from the potential link between low HDL levels and an increased susceptibility to nephrolithiasis, mediated by the influence of reduced HDL on urinary pH and thus facilitating the formation of uric acid-containing kidney stones. The PCs serves as a valuable marker for assessing systemic inflammation. Conversely, HDL-C plays a crucial role in attenuating inflammatory responses. Therefore, PHR could be considered an alternative marker for measuring inflammation, particularly in relation to nephrolithiasis.

Previous epidemiological investigations have supported the link between various risk factors and the likelihood of developing. These risk factors include obesity, smoking, alcohol consumption, DM, hypertension, hyperlipidemia, CKD and CHD. The Taylor trial revealed a correlation between obesity and the development of kidney stones. Men with a BMI of 30 or higher exhibited a 1.33-fold increased risk of nephrolithiasis compared to men with a BMI of 21 to 22.9. Similarly, both elderly and young women in the same BMI category displayed a 1.90-fold increased risk (51). Past studies have examined the impact of body size on urine chemistry, revealing that an increase in BMI may potentially elevate lithogenic risk factors. These factors involve reducing urinary volume and citrate concentrations (52). Another study indicated that middle-aged, hypertensive white males might serve as an indicator for nephrolithiasis, which is characterized by the development of kidney stones and has been linked to increased and prolonged urinary calcium excretion in hypertensive patients (53). Kidney stone disease has also been associated with DM, specifically type 2 DM. This association suggests a beneficial connection between the severity of DM and the occurrence of kidney stones, potentially explainable by the effects of insulin resistance (IR) on urinary pH and the transport of ammonium and calcium into the kidneys (54). In their study, Sur et al. (55) conducted a retrospective analysis of medical histories to investigate the presence of stones in patients with hyperlipidemia. Their results revealed a significant association between statin use, which has cholesterol-lowering effects, and a lower risk of stone formation, suggesting a possible link between hyperlipidemia and nephrolithiasis. Khan et al. (56) explored the influence of various inflammatory markers on stone formation, hypothesizing that renal inflammation could damage epithelial tissue and create favorable conditions for crystallization. Ferraro et al. (57) reported that among women, a history of kidney stones was associated with a modest but statistically significantly increased risk of CHD; however, this association was no significant in men. The findings from the subgroup analysis indicated a beneficial relationship that was consistent across various subgroups when stratified by BMI, smoking status, alcohol consumption, DM, hypertension, hyperlipidemia, CKD and CHD. These results are in line with previous studies conducted on the same issue. Furthermore, except for CKD, where the interaction was significant (*P* for interaction = 0.005), no other clinical variables showed any significant dependency on this association (all *P* for interaction > 0.05). These findings suggest a beneficial relationship that may be applicable across different population settings.

The current inquiry boasts several notable strengths. First, the study utilized data from NHANES, and analyses were conducted with the appropriate NHANES sample weights taken into consideration. Second, confounding covariates were carefully adjusted to enhance the reliability of the results and to facilitate their generalizability to broader populations. Third, due to its cost-effectiveness, simplicity, and the breadth of informative parameters it provides, the inclusion of routine blood examination and blood biochemistry offers considerable potential for the diagnosis and management of nephrolithiasis. Consequently, this approach merits further exploration and detailed analysis.

Nevertheless, it is crucial to acknowledge the inherent limitations of this research. First, the identification of kidney stones was based on personal interviews, introducing the possibility of recall bias, recording errors, interviewer bias. Consequently, some asymptomatic kidney stones that would require physical examination for detection may have been overlooked in the database. Second, the database did not include detailed clinical variables such as personal medication histories or the types of kidney stones, both of which warrant further investigation. Moreover, blood samples were collected from one blood test only. Sequential testing could potentially be more indicative of true physiological states, given the lifespan of blood cells. Third, due to the limitations inherent to the NHANES database, several confounding factors that could influence the results were not considered in the analysis. Fourth, given the randomly missing data among the covariables and the large sample size, the study refrained from employing multiple imputation methods to handle the missing data, which may affect the precision of the findings. However, this approach may potentially impact the accuracy of the findings. Lastly, this study employed a cross-sectional study design, which limits the capacity to determine a causal connection between PHR and nephrolithiasis.

## Conclusion

In summary, our study has demonstrated an independent and non-linear association between PHR and nephrolithiasis, suggesting that PHR could serve as a novel biomarker for predicting the occurrence of this condition and thereby informing individualized therapy and clinical decision-making. However, the establishment of PHR’s reliability as a predictive marker necessitates further validation through comprehensive prospective studies.

## Data availability statement

The original contributions presented in the study are included in the article/**Supplementary Material**. Further inquiries can be directed to the corresponding author. The publicly available datasets presented in this study can be found in online repositories. These data can be found here: https://www.cdc.gov/nchs/nhanes/.

## Ethics statement

The studies involving human participants were reviewed and approved by National Center for Health Statistics Research Ethics Review Board. The patients/participants provided their written informed consent to participate in this study. The studies were conducted in accordance with the local legislation and institutional requirements.

## Author contributions

JN: Conceptualization, Writing – review & editing, Data curation, Formal analysis, Investigation, Methodology, Software, Visualization, Writing – original draft. LL: Data curation, Formal analysis, Writing – original draft. PW: Writing – original draft, Methodology. CX: Conceptualization, Supervision, Writing – review & editing. All authors contributed to the article and approved the submitted version.
