# Supplementary material for: Associations between the platelet/high-density lipoprotein cholesterol ratio and likelihood of nephrolithiasis: a cross-sectional analysis in United States adults
Source: Front Endocrinol (Lausanne). 2024 Feb 21;15:1289553. doi: 10.3389/fendo.2024.1289553 (PMC10914985; doi:10.3389/fendo.2024.1289553)
Supplement: Supplementary file 1 [file Table_1.doc]

| **Supplementary Table 1** **Initial descriptions of participants between with and without nephrolithiasis from the 2007–2018 cycles** | | | | |
| --- | --- | --- | --- | --- |
| **Characteristics (weighted)** |  | **Nephrolithiasis** | |  |
|  | **Total (N = 30899)** | **No (N = 27953)** | **Yes (N = 2946)** | ***P*-value** |
| Age (years) | 47.78±0.23 | 47.13±0.24 | 53.60±0.32 | < 0.0001 |
| PHR continuous | 19.30±0.11 | 19.20±0.11 | 20.20±0.24 | < 0.0001 |
| Platelet (1000cells/uL) | 243.50±0.82 | 243.85±0.80 | 240.39±1.74 | 0.03 |
| TC (mmol/L) | 4.99±0.01 | 4.99±0.01 | 4.97±0.03 | 0.39 |
| HDL-C (mmol/L) | 1.38±0.01 | 1.39±0.01 | 1.29±0.01 | < 0.0001 |
| LDL-C (mmol/L) | 2.94±0.01 | 2.94±0.01 | 2.94±0.03 | 0.92 |
| Fasting glucose (mmol/L) | 5.96±0.02 | 5.92±0.02 | 6.36±0.06 | < 0.0001 |
| Uric acid (umol/L) | 323.20±0.80 | 322.06±0.83 | 333.50±2.05 | < 0.0001 |
| eGFR (ml/min/1.73m^2) | 94.14±0.31 | 94.86±0.33 | 87.60±0.47 | < 0.0001 |
| Creatinine (umol/L) | 78.37±0.26 | 77.84±0.25 | 83.13±0.92 | < 0.0001 |
| Blood urea nitrogen (mmol/L) | 4.93±0.02 | 4.88±0.02 | 5.33±0.06 | < 0.0001 |
| Total plain water (ml/day) | 1155.25±15.62 | 1158.52±16.26 | 1126.21±30.54 | 0.3 |
| CDAI | 0.81±0.05 | 0.84±0.05 | 0.53±0.12 | 0.01 |
| DII | 1.40±0.03 | 1.38±0.03 | 1.58±0.05 | < 0.001 |
| SII (10^9/L) | 534.25±3.49 | 531.11±3.54 | 562.65±9.19 | < 0.001 |
| PHR, n (%) |  |  |  | < 0.0001 |
| T1 (< 15.32) | 10301(33.72) | 9432(34.28) | 869(28.68) |  |
| T2 (15.32–21.36) | 10293(33.63) | 9303(33.58) | 990(34.09) |  |
| T3 (21.36–183.52) | 10305(32.65) | 9218(32.14) | 1087(37.24) |  |
| Age (years, n (%)) |  |  |  | < 0.0001 |
| <45 | 12670(44.72) | 11886(46.35) | 784(30.03) |  |
| 45-64 | 10755(36.48) | 9596(35.68) | 1159(43.77) |  |
| >=65 | 7474(18.80) | 6471(17.98) | 1003(26.20) |  |
| Sex, n (%) |  |  |  | < 0.0001 |
| Male | 15133(48.73) | 13481(48.04) | 1652(54.94) |  |
| Female | 15766(51.27) | 14472(51.96) | 1294(45.06) |  |
| Race, n (%) |  |  |  | < 0.0001 |
| Mexican American | 4706(8.63) | 4321(8.91) | 385(6.15) |  |
| Non-Hispanic Black | 6355(10.68) | 5978(11.23) | 377(5.67) |  |
| Non-Hispanic White | 12785(66.72) | 11181(65.59) | 1604(76.89) |  |
| Other race | 7053(13.97) | 6473(14.27) | 580(11.29) |  |
| Marital status, n (%) |  |  |  | < 0.0001 |
| Married | 18336(63.26) | 16473(62.66) | 1863(68.68) |  |
| Live separated | 6947(18.57) | 6158(18.27) | 789(21.29) |  |
| Never married | 5601(18.13) | 5309(19.04) | 292(10.00) |  |
| Missing | 15(0.03) | 13(0.03) | 2(0.02) |  |
| Education level, n (%) |  |  |  | 0.83 |
| Less than high school | 3295(5.48) | 2970(5.51) | 325(5.21) |  |
| High school | 11336(33.36) | 10247(33.28) | 1089(34.04) |  |
| More than high school | 16233(61.10) | 14703(61.14) | 1530(60.70) |  |
| Missing | 35(0.07) | 33(0.07) | 2(0.05) |  |
| Family PIR, n (%) |  |  |  | 0.18 |
| < 1 | 6038(13.42) | 5475(14.68) | 563(13.08) |  |
| 1-3 | 11936(33.43) | 10750(36.04) | 1186(37.30) |  |
| > 3 | 10023(45.57) | 9078(49.28) | 945(49.62) |  |
| BMI (kg/m^2, n (%)) |  |  |  | < 0.0001 |
| <30 | 8708(29.12) | 8134(30.49) | 574(19.62) |  |
| >=30 | 21800(69.89) | 19470(69.51) | 2330(80.38) |  |
| Smoke status, n (%) |  |  |  | < 0.0001 |
| Never | 17190(55.62) | 15751(56.23) | 1439(50.11) |  |
| Former | 7418(24.66) | 6503(24.04) | 915(30.25) |  |
| Now | 6271(19.68) | 5681(19.68) | 590(19.62) |  |
| Missing | 20(0.04) | 18(0.04) | 2(0.02) |  |
| Alcohol usage, n (%) |  |  |  | < 0.0001 |
| Never | 3950(9.86) | 3590(9.85) | 360(9.94) |  |
| Former | 4296(11.44) | 3752(10.96) | 544(15.78) |  |
| Moderate | 6736(25.45) | 6186(25.71) | 550(23.03) |  |
| Heavy | 5540(19.33) | 5131(19.90) | 409(14.20) |  |
| Missing | 10377(33.92) | 9294(33.57) | 1083(37.05) |  |
| DM, n (%) |  |  |  | < 0.0001 |
| No | 24817(85.27) | 22774(86.42) | 2043(74.89) |  |
| Yes | 6082(14.73) | 5179(13.58) | 903(25.11) |  |
| Hypertension, n (%) |  |  |  | < 0.0001 |
| No | 18021(63.31) | 16716(64.94) | 1305(48.63) |  |
| Yes | 12877(36.69) | 11236(35.06) | 1641(51.37) |  |
| Missing | 1(0.00) | 1(0.00) | 0(0.00) |  |
| Hyperlipidemia, n (%) |  |  |  | < 0.0001 |
| No | 8698(29.29) | 8103(30.22) | 595(20.86) |  |
| Yes | 22201(70.71) | 19850(69.78) | 2351(79.14) |  |
| CKD, n (%) |  |  |  | < 0.0001 |
| No | 24839(84.33) | 22731(85.09) | 2108(77.44) |  |
| Yes | 5665(14.56) | 4860(13.80) | 805(21.41) |  |
| Missing | 395(1.11) | 362(1.10) | 33(1.15) |  |
| CHD, n (%) |  |  |  | < 0.0001 |
| No | 29512(96.25) | 26829(96.59) | 2683(93.16) |  |
| Yes | 1274(3.51) | 1034(3.18) | 240(6.45) |  |
| Missing | 113(0.24) | 90(0.23) | 23(0.40) |  |
| Continuous data are shown as means and standard error (SE), while categorical data are presented as percentages.  **Abbreviations:** LDL-C, low-density lipoprotein cholesterol; HDL-C, high-density lipoprotein cholesterol; TC, total cholesterol; PIR, poverty income ratio; BMI, body mass index; PHR, platelet/high-density lipoprotein cholesterol ratio; CKD, chronic kidney disease; DM, diabetes mellitus; eGFR, estimated glomerular filtration rate; CHD, coronary heart disease; CDAI, composite dietary antioxidant index; DII, dietary inflammation index; SII, systemic immune-inflammatory index. | | | | |
